# The Incremental Cost of Delivering PrEP as a Bridge to ART for HIV Serodiscordant Couples in Public HIV Care Clinics in Kenya

**DOI:** 10.1155/2019/4170615

**Published:** 2019-05-02

**Authors:** Elizabeth M. Irungu, Monisha Sharma, Christopher Maronga, Nelly Mugo, Kenneth Ngure, Connie Celum, Ruanne V. Barnabas, Jared Baeten, Renee Heffron

**Affiliations:** ^1^Centre for Clinical Research, Kenya Medical Research Institute, Nairobi, Kenya; ^2^Department of Global Health, University of Washington, 325 Ninth Avenue, Seattle, WA 98104, USA; ^3^College of Health Sciences, Jomo Kenyatta University of Agriculture and Technology, P.O. Box 62000-00200, Nairobi, Kenya; ^4^Department of Epidemiology, University of Washington, 325 Ninth Avenue, Seattle, WA 98104, USA; ^5^Department of Medicine, University of Washington, 325 Ninth Avenue, Seattle, WA 98104, USA; ^6^Vaccine and Infectious Diseases Division, Fred Hutchinson Cancer Research Center, Seattle, WA, USA

## Abstract

**Background:**

In 2016, the Kenyan Ministry of Health (MOH) released guidelines that recommend preexposure prophylaxis (PrEP) for persons with substantial ongoing HIV risk, including those in HIV serodiscordant partnerships. Estimates of the costs of delivering PrEP within Kenyan public health facilities are needed for planning for PrEP scale up.

**Methods:**

We estimated the incremental annual costs of providing PrEP to HIV uninfected partners as a time-limited “bridge” until the infected partner is virally suppressed on ART within HIV serodiscordant couples as part of routine clinic care in Thika, Kenya. Costs were collected from the Partners Demonstration Project, a prospective evaluation of integrated delivery of preexposure prophylaxis (PrEP) and antiretroviral therapy (ART) to high-risk HIV serodiscordant couples. We conducted time and motion studies to distinguish between activities related to research, routine clinical care, and PrEP delivery. Costs (2015 US dollars) were collected from the MOH perspective and divided into staff, transportation, equipment, supplies, buildings and overhead, and start-up.

**Results:**

PrEP related activities conducted during the screening, enrollment, and follow-up visits took an average of 13 minutes, 51 minutes, and 12 minutes, respectively. Assuming a staff structure of 3 counselors, 1 nurse, and 2 clinicians, we estimate that 3,178 couples can be screened, 1,444 couples offered PrEP and ART, and 6,138 couples followed up annually in an average HIV care clinic. Using costs incurred by the MOH for personnel, drug, and laboratory tests, we estimate that the incremental cost of offering PrEP to HIV uninfected partners within existing ART programs is $86.79 per couple per year. Personnel and PrEP medication made up the largest portion of the costs. We estimate that the total cost to Ministry of Health of delivering integrated PrEP and ART program in public health facilities is $250.19 per HIV serodiscordant couple per year.

**Conclusions:**

Time-limited provision of PrEP to the HIV uninfected partner within HIV serodiscordant couples can be an affordable delivery model implemented in HIV care programs in Kenya and similar settings. These costs can be used for budgetary planning and cost effectiveness analyses.

## 1. Introduction

Despite tremendous gains made towards ending the HIV epidemic, about two million new HIV infections occur each year [1]. The majority of new infections occur in Africa [2] with transmission between members of stable, heterosexual HIV serodiscordant couples (i.e., in which one member is HIV infected and the other uninfected) accounting for an important fraction [3, 4]. In 2012, the national AIDS survey found that, among the 260,000 HIV serodiscordant couples in Kenya, 62% of the HIV infected partners were unaware of their HIV status and only 56% of those on antiretroviral treatment (ART) had achieved viral suppression [5]. More recent estimates indicate that even though all the 1.5 million people living with HIV infection in Kenya are eligible for treatment, only three-quarters are aware of their status and on treatment and of these 63% have attained viral suppression [1].

For HIV serodiscordant couples, ART and preexposure prophylaxis (PrEP) are HIV prevention interventions that significantly reduce the risk of sexual HIV transmission [6, 7]. Mathematical models have projected that high ART coverage can considerably reduce HIV burden [8–10] but some HIV infected persons eligible for ART decline immediate initiation [11]. Additionally, HIV-infected persons require an average of 3-6 months on ART before they achieve viral suppression, leaving an additional gap of time before ART can provide sufficient protection against transmission [7]. As a user-controlled method of HIV prevention, PrEP can fill this gap in protection.

Pragmatic delivery models of antiretroviral-based interventions for HIV serodiscordant couples in resource-constrained settings are necessary to maximize coverage and have an impact on HIV transmission at reasonable costs. One promising model is the use of PrEP as a “bridge” to sustained ART use. This is time-limited provision of PrEP to the HIV uninfected partner when the HIV infected partner is not yet on ART or while on ART but not yet virally suppressed. When viral suppression is achieved, protection against transmission within the partnership is provided by sustained ART use by the HIV infected partner and PrEP is no longer necessary, in the absence of outside partnerships. Previous studies of HIV serodiscordant couples have reported that about 30% of incident HIV infections are most likely from outside partnerships [12, 13]. Delivery of PrEP as a “bridge” to ART among HIV serodiscordant couples in East Africa was found to be highly effective and cost-effective [14–16]. The World Health Organization and the Kenyan Ministry of Health now recommend provision of PrEP and ART for HIV serodiscordant couples in this manner [17, 18]. An important consideration for policy makers charged with implementing integrated PrEP and ART for couples is the cost of resources needed for delivery. We provide estimates of the cost of delivering antiretroviral-based HIV prevention to HIV serodiscordant couples in public health facilities in Kenya and the incremental cost of providing PrEP as a component of this strategy.

## 2. Methods

### 2.1. The Partners Demonstration Project

The Partners Demonstration Project was an open-label interventional study, aimed at modeling real-world delivery of an integrated PrEP and ART program to high-risk serodiscordant couples conducted in four sites in Kenya and Uganda between November 2012 and June 2016 [14]. Across all sites, 1694 couples were screened and 1,013 enrolled. At the site in Thika, Kenya, the screen-to-enroll ratio was 2.2 and 332 couples were enrolled. Retention was above 85% throughout follow-up [19]. HIV infected partners initiated ART according to their countries' national guidelines. HIV uninfected partners were offered daily oral combination emtricitabine/tenofovir disoproxil fumarate (FTC/TDF) as PrEP which was recommended to be discontinued approximately six months after ART initiation by the HIV infected partner when viral suppression was expected. Thus, we implemented time-limited PrEP as a bridge to ART and viral suppression.

At the screening visit, to determine eligibility, demographic and behavioral information were collected and laboratory tests were conducted including HIV testing for both partners, serum creatinine and hepatitis B surface antigen (HBsAg) for HIV uninfected partners, and CD4 count and plasma HIV RNA concentrations for HIV infected partners. Couples were eligible if they were sexually active and intended to remain in the partnership. Couples in which the HIV infected partner was using ART or had WHO stage III or IV were excluded. Higher risk couples were eligible if they had a risk score ≥5 on an empiric risk score for HIV transmission from previous cohorts of HIV serodiscordant couples [20, 21]. At enrollment, couples received counselling on risk reduction and benefits of PrEP and ART. PrEP was offered to the HIV uninfected partner. ART was initiated according to Kenyan national guidelines. Follow-up visits were scheduled 1 month after enrollment and then quarterly for up to 24 months. Couples were encouraged to come to the clinic together, whenever possible. At the follow-up visits, HIV testing was conducted for the HIV uninfected partner, PrEP, and ART adherence counseling and provision of prescriptions was done as needed. At the outset of each visit, couples were seen by a HIV counsellor and then a clinician for clinical review and prescription of medications as needed.

### 2.2. Time and Motion Observation

At the Thika, Kenya site, we conducted time and motion observation of intervention activities over 13 weeks (6^th^ March 2014–29^th^ May 2014) to determine the additional time required to conduct PrEP-related activities and estimate the amount of time required for one couple to complete a PrEP visit. The observation was conducted by clinical research staff whose familiarity with project procedures and local languages aided in data quality. When study participants arrived at the clinic, a staff member would use a stop watch to determine the length of their activities and note the start and end times of each activity (e.g., registration at the reception desk, waiting in the reception area, and counseling). If a couple arrived together but completed some visit procedures separately, the staff member would follow one of the members of that partnership. Periods with inactivity were recorded as waiting time. Time and motion observation was conducted until we reached saturation of information. We observed visits conducted at varying times of day and by different staff members to obtain a representative sample of observations.

The activities were placed in three mutually exclusive categories: research, routine clinical care, and PrEP-related. Research activities included administering informed consent and updating any demographic information or contact details. Routine clinical care activities were those considered to be normal standard of care activities that are performed in HIV care and treatment programs, such as ART adherence counselling and general risk reduction counselling. The remaining time costs were PrEP-related activities and included most activities for the HIV uninfected partner, such as PrEP medication adherence counselling and prescription dispensing. Data from the time and motion studies were used to estimate the average number of couples that could be seen in a typical clinic annually by applying average visit lengths to a range of couples that could be seen per day and extrapolating this to one year while accounting for meetings and other staff activities.

### 2.3. Microcosting

We conducted activity based microcosting in 2015 US dollars from the payer perspective following established guidelines [22, 23]. Supplementary cost data were collected by (1) interviewing staff to assess concordance with results from our time in motion studies, (2) examining study budgets and invoices, and (3) interviewing local experts to corroborate information on estimates of Ministry of Health costs of supplies, commodities, and salaries. All the capital, start-up, and training costs were annualized assuming 5 years of useful life with a 3% annual discount rate. We assumed staff would work an average of 231 days per year (accounting for weekends, national holidays, and standard vacation allowances). Total annual program costs were divided by the number of couples initiating PrEP to determine the annual intervention cost per couple. All costs were divided into seven mutually exclusive categories: personnel, recruitment activities, start-up, building, laboratory tests, medication, and supplies.

We estimated costs of integrated PrEP and ART delivery to HIV serodiscordant couples for 3 scenarios: (1) an as-studied scenario, (2) a Ministry of Health scenario, and (3) an as-studied scenario without research components (see supplement available here). The Ministry of Health scenario is the most relevant for programmatic scale-up of PrEP and ART delivery for HIV serodiscordant couples while the other two represent upper bounds of the costs.

### 2.4. As Studied Scenario

This scenario reflects the cost of conducting the project exactly as it was done at the research clinic. Personnel costs included the annual salaries of the research staff delivering the intervention. Recruitment activities included conducting site visits to referral clinics, couple pick-ups, twice-a-month visits to local clinics by outreach staff to conduct seminars on PrEP and ART, development of informational material, and community activities to promote couples' HIV counselling and testing. Start-up costs included a 3-day off-site protocol training for all the project staff and development of standard operating procedures. The laboratory costs included HIV testing (for both members of the couple at baseline and for the HIV uninfected partner at each visit), 6-monthly plasma HIV RNA concentrations, and creatinine testing at month 1 and every 6 months. For this scenario the cost of FTC/TDF was obtained from the local pharmacies while that of ART was obtained from the 2015 Clinton Health Access Initiative (CHAI) reference price list [24].

### 2.5. Ministry of Health Scenario

This scenario reflects the expected program costs if the intervention were implemented by the Kenyan Ministry of Health in public HIV care clinics. Public HIV care clinics serve the majority of HIV infected people in Kenya. They provide regular clinical and laboratory monitoring and provide antiretroviral medications and other treatments for opportunistic infections. The clinics are typically run by clinical officers, nurses, HIV counsellors, pharmacy personnel, and laboratory personnel [25]. To obtain government personnel costs, we revised the project staff structure to reflect that of a typical HIV care and treatment facility (3 HIV testing and counselling service [HTS] providers, 2 clinical officers, 1 nurse counsellor, 1 pharmaceutical technologist, and 1 laboratory technician). Health workers were assumed to work five days a week for seven hours a day, after accounting for a one-hour lunch break. We replaced research-level staff salaries with public sector salaries [26]. We excluded visits to referral clinics and providing transportation for couples to the clinic and community activities. Thus, the recruitment activities cost for this scenario was development of informational material and counsellor-led seminars, which were reduced from bimonthly to monthly sessions. The length of the training was reduced to two days after removing research related components. Additionally, the number of staff attending the training and the training venue costs were revised to reflect those of a typical government training event. Costs of laboratory tests were revised to reflect those incurred in public health facilities [27]. The frequency of serum creatinine testing was reduced to once per year for the HIV uninfected partner on PrEP, while plasma HIV RNA concentrations were done at 6 and 12 months as recommended by the Kenyan national guidelines [17]. We used the CHAI lowest negotiated annual prices of TDF/FTC estimated at $67, while that of ART (tenofovir, lamivudine, and efavirenz, as a fixed dose combination, which is the first line regimen in Kenya) was estimated at $110.40 [24]. We consulted with government experts to obtain estimates of quantity of supplies required, which included stationery and clinic consumables such as gloves, needles, and vacutainers and used government price lists to estimate the costs [28, 29].

We conducted sensitivity analysis to assess how the cost of PrEP delivery changes with varying programmatic assumptions such as the length of time on PrEP. We also conducted sensitivity analyses to assess how the incremental cost of PrEP delivery changes with varying screening and enrollment times.

### 2.6. Ethics Statement

The University of Washington Human Subjects Review Committee and ethics review committees at collaborating institutions at each of the study sites (Kenya Medical Research Institute, Kenyatta National Hospital and Uganda National Council of Science and Technology) approved the study protocol. All participants provided written informed consent in English or in their local languages.

## 3. Results

### 3.1. Time in Motion Study

We observed and timed 18 visits: 1 screening that lasted 2.1 hours, 2 enrollment visits, with an average time of 2.7 hours, and 15 follow-up visits that lasted 1.2 hours on average. (Table 1). Excluding the research components, the time taken to conduct activities related to delivery of ART and PrEP for the HIV serodiscordant couples was 42 minutes at screening visits (33%), 66 minutes at enrollment visits (41%), and 36 minutes during follow-up visits (51%), respectively. Specifically, PrEP related activities took 13 minutes (31% of time related to ART and PrEP delivery), 51 minutes (77%), and 12 minutes (33%), respectively. The time spent by a couple with an HIV counsellor was 35 minutes, 28 minutes, and 19 minutes at the screening, enrollment, and follow-up visits, respectively. There were seven visits in which a couple arrived together but completed some visit procedures separately. The HIV infected partner was followed in 4 of the visits and the HIV uninfected partner in 3 of the visits. The times observed for these separated visits were comparable to those observed when participants came to the clinic alone.

We estimate that 3 HIV counsellors in a Ministry of Health clinic that provides services for HIV serodiscordant couples only and has a screen to enroll ratio of 2:2 and an 85% retention rate can assess 3,178 couples for PrEP eligibility, enroll 1,444 eligible couples into an integrated PrEP and ART program, and conduct 6,138 follow-up visits annually. Thus, one counsellor or clinician working in a public health HIV care clinic can enroll 25 HIV serodiscordant couples into care and conduct up to 107 follow-up visits in a month.

### 3.2. Microcosting

**Figure 1:**
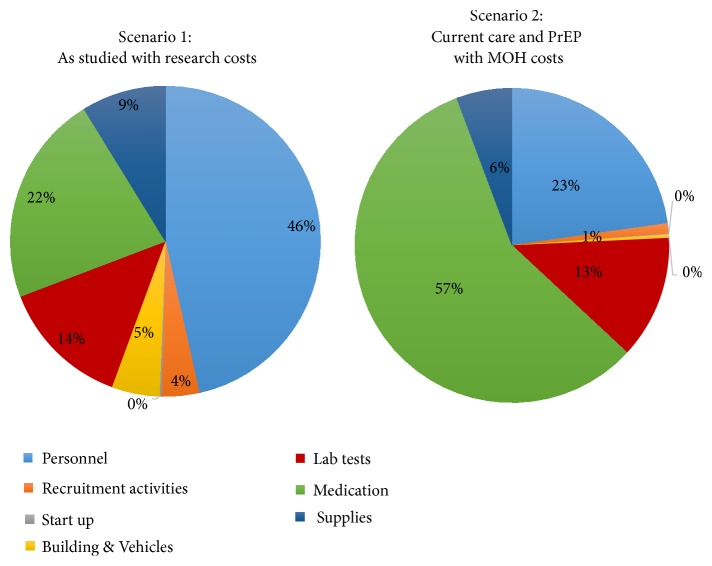
Proportion of costs related to each resource type of a program to deliver integrated PrEP and ART to HIV serodiscordant couples.* The personnel cost is the salary for personnel providing service, recruitment activities include costs of activities that helped identify HIV serodiscordant couples and brought them to the study site including, information, education, and communication (IEC) material and support supervision visits to HIV testing and counselling staff to encourage couple testing in order to identify HIV serodiscordant couples, start up: costs associated with training and development of standard operating procedures, building: cost of rent, laboratory: costs of performing tests for PrEP delivery, including HIV and creatinine tests, medication: costs of PrEP and ART; supplies: costs of office stationery and clinical consumables.*

**Figure 2:**
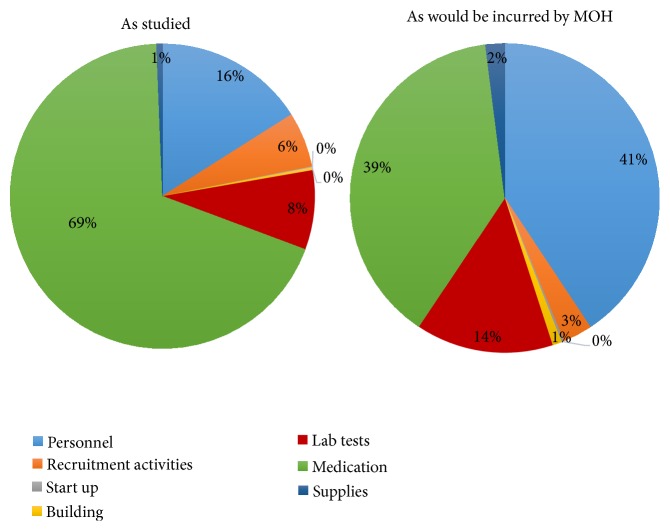
Allocation of incremental cost of PrEP delivery in a year, by resource type.* The personnel cost is the salary for personnel providing service, recruitment activities include costs of activities that helped identify HIV serodiscordant couples and brought them to the study site including information, education, and communication (IEC) material and support supervision visits to HIV testing and counselling staff to encourage couple testing in order to identify HIV serodiscordant couples, and start-up costs are costs associated with training and development of standard operating procedures; building, cost of rent; laboratory, costs of performing tests for PrEP delivery, including HIV and creatinine tests; medication, costs of PrEP; and supplies, costs of office stationery and clinical consumables.*

The cost of delivering the full “PrEP as a bridge to ART” strategy in the as-studied scenario was $1454.87 per couple per year (Table 2). Using government pricing, the cost would have been $250.19 per couple per year. The costs of personnel, medication, and laboratory testing accounted for most of the cost differences across the two scenarios (Figure 1). The incremental cost of delivering the PrEP component of the strategy to HIV serodiscordant couples was $305.75 per year in the as studied scenario and $86.79 when Ministry of Health costs were applied (Table 3). If PrEP use was extended to 9 months to account for potential delays in ART initiation by the infected partner, longer time to viral suppression associated with nonadherence, or presence of outside partnerships, the incremental cost of PrEP incurred by the government would be $103.54, an increase of 19.3% due to the additional 3 months of drugs. Each additional month of PrEP use resulted in a 6.4% rise in the incremental cost of PrEP delivery under government costs. Personnel and medication costs accounted for more than eighty percent of the incremental costs required for delivery of the full strategy, while laboratory testing accounted for 14% of the additional costs (Figure 2).

### 3.3. Sensitivity Analysis

The incremental cost of PrEP was $100.26 if the screening time was doubled and $80.06 if the screening time was halved. Similarly, if the time taken to conduct enrollments was doubled or halved, the incremental cost of PrEP was $91.50 and $84.44, respectively. If both screening and enrollment times were doubled or halved, the incremental cost would be $104.97 and $77.70, respectively.

## 4. Discussion

Delivery of PrEP to HIV uninfected partners integrated with ART provision to the HIV infected partners among HIV serodiscordant couples in Kenyan public health facilities can be affordable. Our estimates of the cost to the Ministry of Health is $250.19 per couple per year, which includes the funding for the HIV care and ART programs that the government already provides to the HIV infected partner. ART program costs in Kenya are estimated to be between $181 and $256 per year [30]. Thus, we have estimated that the incremental cost is $87 for the government HIV care programs to add time-limited PrEP for the HIV uninfected partner in a serodiscordant partnership. These results, though specific to the Kenyan context, approximate those found in a microcosting study of the Partners Demonstration Project of integrated PrEP and ART conducted at a Ugandan site [16].

We identified areas to improve efficiency in the delivery of the intervention. In the Partners Demonstration Project, couples were screened out because they did not meet the risk score criteria, which were meant to identify couples at highest risk of acquiring HIV. This resulted in a high screen to enroll ratio which may increase screening costs, but may also improve cost-effectiveness of the intervention. To increase efficiency, programs can conduct demand creation activities with tailored messages to encourage couples who are at high risk for HIV transmission to access PrEP services. In our study, laboratory assessments related to eligibility, such as creatinine and HBsAg testing, and PrEP initiation for the eligible couples were done on separate visits. However, in national roll out, these activities are conducted on the same day, reducing time and costs of PrEP initiation. In addition, in the Kenya PrEP guidelines, PrEP can be initiated without these tests, if they are unavailable. Use of lower cost FTC/TDF generics may further reduce the cost.

The major limitation of our study is that cost estimates were obtained from a program delivered in a research setting in Thika, Kenya, via clinicians normalized to clinical research and there may be elements that do not reflect the situation in a public facility (e.g., staff highly trained in PrEP, more supervision). However, we adjusted our analysis to reflect costs and time associated with patient visits to a government facility. It is possible that the intervention would take longer or have lower effectiveness if run by less experienced staff. To address this uncertainty, the higher costs estimated from the as-studied and as-studied without research scenarios may serve as a guide to the upper bound of potential costs that would be incurred when delivery is done in a less efficient system. We do not include scale-up costs needed to reach peak program efficiency. For example, when the national PrEP program began, health providers took a long time to see clients and provide the intervention, which is associated with higher costs, but time needed for the intervention is expected to reduce as providers are becoming familiar with activities associated with counseling for and prescribing PrEP [31]. Another limitation of this study is that we observed only one eligibility assessment visit. However, the duration of that visit was comparable to the duration reported in the microcosting study conducted in Uganda [16] and in interviews with study staff about time spent conducting eligibility assessment visits. Finally, we assumed a set of recruitment activities were conducted to generate demand for the intervention. If recruitment effectiveness or the set of recruitment activities implemented is different when conducted as a government program, costs incurred may be different from what we projected. Future studies are needed to assess the incremental cost of this intervention when conducted in Ministry of Health clinics.

To our knowledge this is the first study to assess the costs of delivering PrEP to HIV uninfected members of HIV serodiscordant couples in Kenya. Our findings are timely and relevant since Kenya has initiated national roll out of PrEP for HIV serodiscordant couples and all persons with substantial on-going risk of HIV infection and these cost estimates will help guide resource mobilization and allocation for this novel intervention. Time-limited provision of PrEP to HIV uninfected partners and effective ART use by the HIV infected partner is a very effective delivery approach and potentially affordable on a large scale to have high coverage and impact to prevent HIV transmissions in Kenya and in other African countries with a generalized HIV epidemic.

## Figures and Tables

**Table 1:** Average time (in hours) required to conduct intervention visits by type and activity.

Visit type	Number of observed visits	Research-related activities	Current care activities	PrEP-related activities	All activities
Screening	1	1.42	0.48	0.23	2.13

Enrollment	2	1.56	0.25	0.86	2.67

Follow up	15	0.56	0.40	0.20	1.15

**Table 2:** Annual cost (2015 USD) of delivering integrated PrEP and ART to HIV serodiscordant couples.

Type of cost	Scenario 1: As-studied with research costs	Scenario 2: Substituting Ministry of Health costs
Number of enrolled couples per scenario	*521*	*1444*

Total cost	*$757,483.58*	*$361,304.58*

Personnel	$352,156.45	$82,200.00

Medication	$166,608.84	$207,235.34

Laboratory tests	$103,094.95	$45,825.24

Supplies	$66,587.00	$20,651.04

Building and Vehicle costs	$37,769.21	$1,200.00

Recruitment activities	$29,527.00	$3875.00

Start up activities	$1,740.13	$317.97

Cost per couple	*$1,454.87*	*$ 250.19*

*∗*Medication and laboratory costs increase since the number of clients that can be attended to increases when the research component is excluded.

**Table 3:** Annual incremental cost of adding a PrEP component for HIV uninfected partners in HIV serodiscordant relationships to the current ART program.

Type of cost	As studied less research and current care costs		With Ministry of Health costs	
	For 1444^*∗*^ couples	Per couple	For 1444^*∗*^ couples	Per couple

Personnel	$70,723.85	$48.97	$51,000.00	$35.31

Medication	$303,271.22	$210.00	$48,378.98	$33.50

Laboratory tests	$37,186.83	$25.75	$18,051.86	$12.50

Supplies	$2,793.72	$1.93	$2,514.35	$1.74

Building and Vehicle costs	$1,200.00	$0.83	$1,200.00	$0.83

Recruitment activities	$25,957.00	$17.97	$3,875.00	$2.68

Start up activities	$422.77	$0.29	$317.97	$0.22

Total cost (US $)	*$441,555.40*	*$305.75*	*$125,338.15*	*$86.79*

^*∗*^We estimate that an HIV care clinic can enroll 1444 HIV serodiscordant couples into the integrated PrEP and ART program in a year.

## Data Availability

The data used to support the findings of this study are included within the article.

## Supplementary Materials

Supplementary Materials Supplementary Document 1: description and results of the as-studied scenario without research components.
