# Supplementary material for: The Incremental Cost of Delivering PrEP as a Bridge to ART for HIV Serodiscordant Couples in Public HIV Care Clinics in Kenya
Source: AIDS Res Treat. 2019 May 2;2019:4170615. doi: 10.1155/2019/4170615 (PMC6521338; doi:10.1155/2019/4170615)
Supplement: Supplementary Materials — Supplementary Document 1: description and results of the as-studied scenario without research components. [file 4170615.f1.pdf]

The incremental cost of delivering PrEP as a bridge to ART for HIV serodiscordant couples in public HIV care clinics in Kenya

As studied scenario without research component

This scenario reflects the costs that would be incurred by programs, such as privately-run entities or other PrEP program implementing partners that wish to implement PrEP in the same fashion as we did in the research study with a goal of accruing many couples within a short period of time. The costs for this scenario exclude the administrative study personnel and those exclusively involved in research activities, such as the staff conducting quality assurance and quality control for the case reports forms. Recruitment activities such as visits to clinics and conducting PrEP and ART seminars, development of informational material and community activities were included. However, we excluded costs of driving couples to the research clinic and other research related vehicle costs. Building rental costs were excluded but building maintenance costs were included.

Results

The cost of delivering "PrEP as a bridge to ART" strategy in the as-studied scenario without research components was $666.16 per couple per year (Supplement Table).

Supplement Table: Annual cost (2015 USD) of delivering integrated PrEP and ART to HIV serodiscordant couples

Type of cost	Scenario 1: As-studied with research costs	Scenario 2: Substituting Ministry of Health costs	Scenario 3: Current care & PrEP costs (removing research costs)
**Number of enrolled couples per scenario**	**521**	**1444**	**1444**
**Total cost**	**$757,483.58**	**$361,304.58**	**$962,032.84**
**Personnel**	$352,156.45	$82,200.00	$155,851.45
**Medication**	$166,608.84	$207,235.34	*$462,127.58

**Laboratory tests**	$103,094.95	$45,825.24	*$285,957.32
**Supplies**	$66,587.00	$20,651.04	$25,813.80
**Building and Vehicle costs**	$37,769.21	$1,200.00	$4,589.74
**Recruitment activities**	$29,527.00	$3875.00	$26,797.00
**Start up activities**	$1,740.13	$317.97	$895.95
**Cost per couple**	**$1,454.87**	**$ 250.19**	**$666.16**

*medication and laboratory costs increase since the number of clients that can be attended to increases when the research component is excluded.

Supplement Figure: Proportion of costs related to each resource type of a program to delivery integrated PrEP and ART to HIV serodiscordant couples

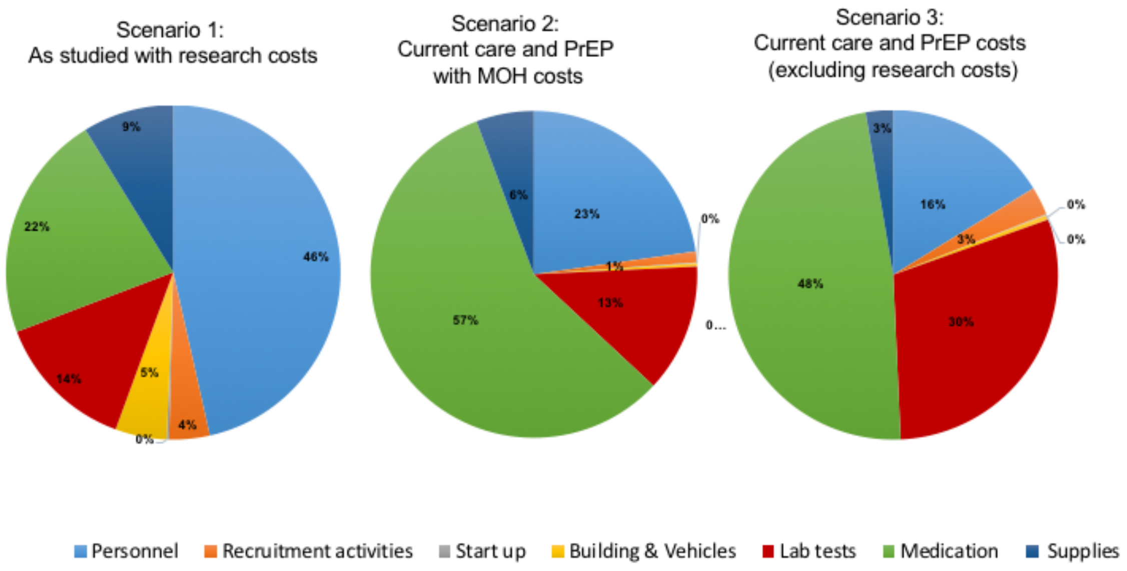

The personnel cost is the salary for personnel providing service, recruitment activities include costs of activities that helped identify HIV serodiscordant couples and brought them to the study site including, information, education and communication (IEC) material and support supervision visits to HIV testing and counselling staff to encourage couple testing in order to identify HIV serodiscordant couples, start up: costs associated with training and development of standard operating procedures, building: cost of rent, laboratory: costs of performing tests for PrEP delivery, including HIV and creatinine tests, medication: costs of PrEP and ART; supplies: costs of office stationery and clinical consumables.
